# Correlation research on physical activity and executive function in female college students with subclinical depression

**DOI:** 10.3389/fpsyt.2024.1403471

**Published:** 2024-05-21

**Authors:** Ping Li, Majed M. Alhumaid, Haiyan Wang, Hai Li, Shanguang Zhao

**Affiliations:** ^1^ Department of Psychiatry, The Second People’s Hospital of Neijiang, Neijiang, China; ^2^ Department of Physical Education, College of Education, King Faisal University, Al-Ahsa, Saudi Arabia; ^3^ School of Foreign Languages, Shanghai Jian Qiao University, Shanghai, China; ^4^ College of Sport, Neijiang Normal University, Neijiang, China; ^5^ Department of Physical Education, Shanghai Maritime University, Shanghai, China

**Keywords:** subclinical depression, physical activity, executive function, female, college students

## Abstract

Researchers have found that there may be a correlation between physical activity, executive function, and depression for college students with depression. However, there is limited information available regarding the relationship and interaction between subclinical depression, physical activity, and executive function among college students with subclinical depression. The purpose of this study is to assess the correlation and interaction between subclinical depression, physical activity, and executive function in female college students with subclinical depression. The ActiGraph GT3X was utilized to measure physical activity time, and the colour-word Stroop task was employed to evaluate the executive function of the participants. The findings revealed that female college students with subclinical depression had a significantly lower time for moderate-intensity physical activity compared to healthy female college students. Additionally, the subclinical depression group took significantly longer to complete the colour-word Stroop task compared to the healthy group of female college students. The results of correlation and mediation analyses suggest a negative correlation between BDI-II scores and physical activity time and executive function in female college students with subclinical depression. Moreover, executive function appears to play a partial mediating role in the relationship between physical activity and subclinical depression.

## 1 Introduction

Depression involves experiencing a depressed mood or loss of pleasure or interest in activities for extended periods of time (1, 2). The World Health Organization (WHO) estimates that approximately 5% of adults worldwide suffer from depression, with a higher prevalence among females than males (2). Depression is a significant concern among college students that should not be overlooked. A 2022 survey assessing the mental health of nearly 80,000 college students in China revealed a depression risk detection rate of approximately 21.48%. Similarly, a 2021 survey in England reported that 37% of undergraduate students were experiencing major depressive symptoms, and a study focusing on international postgraduate students studying public health at a university found a depression prevalence of 34.6% (3, 4). Depression can have a profound impact on various aspects of life, including relationships with family, friends, and community. Among college students, issues such as poor academic performance and low self-esteem can contribute to depression, and in turn, depression can worsen these problems (2, 5). Several studies suggest that depression can have a negative impact on the utilisation or performance of executive function (1, 6).

The executive function, a high-level cognitive processes that regulates thoughts and actions, particularly in nonroutine situations, plays a crucial role in individual learning and problem-solving (7, 8). The core executive functions include: (1) working memory, which involves holding information in mind and mentally processing it; (2) inhibitory control, which involves managing attention, behaviour, thoughts and emotions to overcome internal impulses or external distractions and engage in more appropriate or necessary actions; and (3) cognitive flexibility, which involves shifting perspectives or approaches to problems and adapting flexibly to new demands, rules, or priority events (9, 10). Reasoning, problem-solving, and planning are considered high-level executive functions (10). The difference between core executive functions and high-level executive functions lies in the task requirements (10). Therefore, executive functions are integral to the high-level neurocognitive processes that regulate thoughts and behaviours to achieve goals and manage behavioural, cognitive, and emotional activities through adaptive capacities (11). Executive function is crucial for college students, and its decline can weaken emotional regulation and impact daily life and learning efficiency, including intelligence, academic performance, fatigue, and education level-related issues (12, 13).

However, the relationship between depression and executive function remains a topic of intense debate among researchers, particularly regarding whether executive function is impaired in individuals with depression. Grant et al. (14) study found no significant impairment in executive function among patients with depression. In contrast, Joormann et al. (15) suggest that difficulties in executive function may serve as a precursor to depression and increase the risk of developing it. Additionally, the relationship between depression and specific submodules of executive function, such as inhibitory control, working memory, and cognitive flexibility, has yielded conflicting results. Regarding inhibitory control, Lewis et al. (16) conducted a study involving 2,328 18-year-old participants using a Go-Nogo task. Their findings indicated that impaired inhibitory control may not be indicative of impaired executive function in adolescents with depression. The study observed that the severity of depressive symptoms did not correlate with the error rate of inhibitory control at the one-year follow-up. These results contradict Joormann et al. (15) suggestion that difficulty in emotional inhibition may serve as a precursor to depression. In terms of working memory and cognitive flexibility, some studies have suggested that individuals with depression exhibit impaired attentional control over negative events, which can impact performance in working memory tasks and cognitive flexibility (17, 18). Chuang et al. (19), utilising the Go-Nogo task, found that adolescents with depressive disorders exhibited longer reaction times to negative expressions compared to healthy controls, and this effect was related to age. On the other hand, Fukuzaki and Takeda (20) suggested that cognitive flexibility is relatively stable and not significantly affected by age. Their study found no association between cognitive flexibility, depressive status, or work performance in healthy young workers. Moreover, numerous epidemiological studies have demonstrated a dose-response relationship between physical activity and depression. Research indicates that individuals with depression may reduce their physical activity time, and regular physical activity can effectively prevent and alleviate depressive symptoms (21–23).

However, the relationship between executive function, physical activity, and subclinical depression remains unclear. Subclinical depression refers to a condition in which a person exhibits depressive symptoms that do not meet the criteria for a clinical diagnosis of depression (24). While not as severe as depression, subclinical depression can significantly impact an individual’s quality of life and increase the risk of developing depression later in life (24–27). Studies have shown that students with subclinical depression are twice as likely to experience depression compared to non-depressed students (26). Currently, research rarely explores the relationship between executive function, physical activity, and subclinical depression among college students. Therefore, it is uncertain whether physical activity time and executive function are affected in college students with subclinical depression compared with healthy college students. Additionally, there is limited information on the relationship between physical activity, executive function, and depression in college students with subclinical depression. Differences in biological factors, including hormone levels, genetic inheritance, and social roles and pressures, contribute to a higher prevalence of depression in females compared to males. This gender disparity in depression is further exacerbated in the aftermath of the COVID-19 pandemic (2, 28, 29). Therefore, this study focuses specifically on female college students with subclinical depression. The hypothesis of the study is that there is a correlation between the severity of their symptoms and both physical activity and executive functioning among female college students with subclinical depression. Furthermore, the study proposes that executive function may partially mediate the relationship between physical activity and subclinical depression. The aim is to investigate the relationship between physical activity, executive function, and subclinical depression by comparing female college students with subclinical depression to healthy female college students.

## 2 Methods

This study consisted of three main sections: recruiting and screening participants with subclinical depression and healthy controls, collecting data on physical activity time and the behavioural performance of an executive function, and conducting data analysis.

### 2.1 Participants

First-year female undergraduate students at Shaanxi Normal University in China were invited to participate in this study. Two groups of participants, namely the subclinical depression group and the healthy control group, were identified through screening process among the voluntarily enrolled students. Subclinical depression was screened using two steps. Initially, the Beck Depression Inventory–II (BDI-II) (30) was administered for the initial screening. Subsequently, psychologists from the University Counseling Center conducted interviews based on the DSM-IV to confirm that participants met the criteria for subclinical depression. Therefore, two inclusion criteria were used for screening the subclinical depression group: BDI-II scores between 14 and 27 (31), and confirmation of subclinical depression by psychologists. BDI-II scores less than 13 were the inclusion criteria for the healthy control group. The following people were excluded from the subclinical depression and healthy control groups: (1). Those with colour blindness. (2). Had a prior history of depression or traumatic brain damage. (3). Previously attempted suicide. (4). We’re taking mental health drugs like benzodiazepines, antidepressants, mood stabilizers, or antipsychotics. (5). Possessing medical conditions such as cancer or cerebrovascular disease. (6). Possessing mental health conditions such as post-traumatic stress disorder, bipolar illness, or schizophrenia.

**Figure 1:**
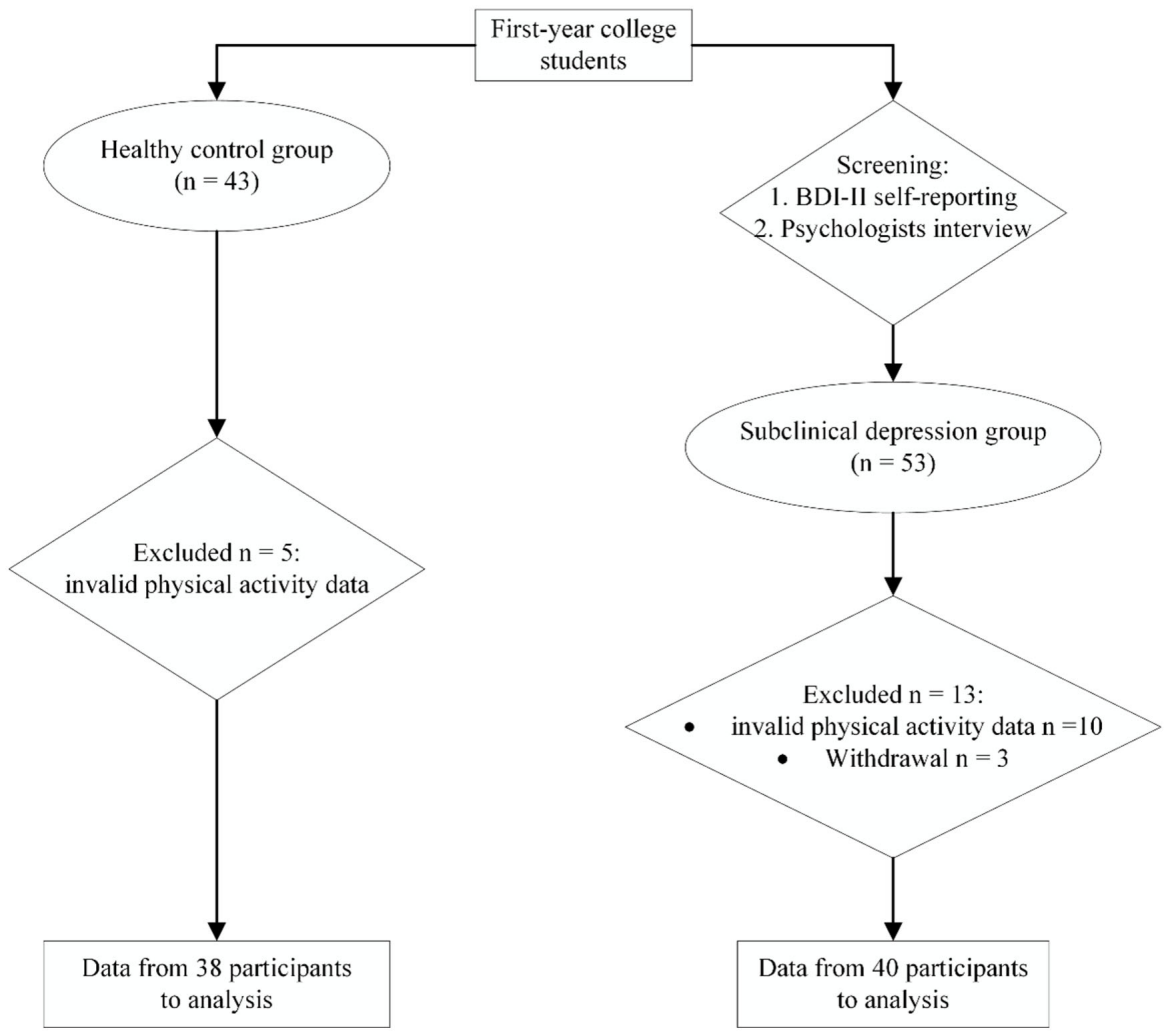
Flow diagram of participant recruitment..

Based on the Edinburgh Handedness Inventory, all participants were identified as right-handed (32) and they possessed adequate visual and auditory capabilities to successfully complete the necessary experiments. The flowchart illustrating participant recruitment is presented in **Figure 1**.

From **Figure 1**, it can be observed that a total of 53 female college students with subclinical depression and 43 healthy female college students were recruited. All participants were granted written informed consent and were reimbursed financially for their involvement. This study obtained research ethics approval from the Ethics Committee of Shaanxi Normal University (202116010).

At the end of the experiment, thirteen participants in the subclinical depression group were excluded: ten due to invalid physical activity data and three due to withdrawals. Additionally, five participants in the healthy control group were excluded due to invalid physical activity data. Therefore, data from 40 participants in the subclinical depression group and 38 in the healthy group were used for the final analysis. A sample size of 26 individuals per group would be adequate to detect a significant impact (*Cohen’s d* = 1.00) with 0.80 power and 0.05 Type I error rate, according to an *a priori* power analysis for independent samples t-test.

### 2.2 Physical activity

The ActiGraph GT3X is a triaxial accelerometer used to record participants’ activity counts. The Actilife 6.1 software was utilised for parameter setting and data analysis of the accelerometer. Prior to the experiment, the accelerometer was initialised, and participants’ height (inches), weight (lb), and age were entered into the device. The accelerometer should be worn on the left wrist, with the accelerometer button-like interface cover facing towards the torso when worn. The accelerometer records data at a time interval (epoch) of 60 seconds, and a minimum of 8 hours of wear time per day is considered as one valid day) (33). To ensure reliable screening of physical activity data, a minimum standard of at least three valid days per week is maintained (34).

The formula chosen for calculating physical activity energy was the Freedson VM3 Combination (35). The Freedson VM3 cut points were used to define energy expenditure for physical activity at moderate and vigorous intensities, specifically above 2,690 counts/min for moderate intensity and above 6,167 counts/min for vigorous intensity (36). Two methods were employed in this study to determine the time spent in daily activities at moderate and vigorous intensities: accumulated in minutes and using the ‘bout’ parameter, which calculates moderate times lasting at least 10 minutes. This aligns with the recommendation of the American Academy of Sports Medicine, which advocates for more than 10 minutes of activity to accrue health benefits (37).

Based on the moderate-intensity physical activity times and the ‘bout’ parameters provided by the accelerometer, the participants’ physical activity level was evaluated according to the WHO physical activity guidelines. According to the guidelines, adults aged 18–64 years are recommended to participate in either 150–300 minutes of moderate-intensity or 75–150 minutes of vigorous-intensity physical activity per week (38).

### 2.3 Executive function

All participants in the study performed the colour-word Stroop task using E-prime 3.0 to measure their behavioural performance of executive function (39). Seated in front of a computer, participants were instructed to respond only to the colour of the words that appeared on the screen, disregarding their actual meaning. Regardless of the word being presented, their responsibility was to rapidly and adequately determine the colour of the text. The reaction time and accuracy of the participants were recorded by E-prime.

In this study, the colour-word Stroop task employed blue, green, and red letters arranged randomly in their corresponding colours of blue, green, and red on a computer screen. Two types of stimulus tasks were presented: consistent stimulus tasks, where the colour and word matched (e.g., the word ‘green’ written in green), and inconsistent stimulus tasks, where colour and word mismatches occurred (e.g., the word ‘green’ written in red). Participants were instructed to respond by pressing the M key with their right index finger for consistent conditions and the Z key with their left index finger for inconsistent conditions.

The colour-word Stroop task comprised both practice and formal experimental phases. The practice phase consisted of eight trials, with participants receiving feedback after each trial regarding the accuracy of their responses. Following the practice phase, the experimenter inquired whether participants fully comprehended the task. If participants were unclear, the practice phase could be repeated until they had a clear understanding of the task. The formal experimental phase consisted of a total of 240 trials. In each trial, consistent and inconsistent conditions appeared randomly, with a ratio of 3:1 for inconsistent to consistent conditions. Each trial, began with the presentation of white fixation points (+) on the screen, displayed in Courier New font, size 24, for 500 ms, with a black background. The interval between the stimulus and fixation point was 300 ms to eliminate any expected effects on the participants. The duration of the colour word stimulus was 1500 ms. After the stimulus disappeared, participants were required to press the button to respond within 1000 ms. Following the button press, there was an interval of 800–1200 ms before the next fixation point appeared.

### 2.4 Data analysis

The data’s normal distribution was assessed through the application of the Shapiro-Wilk test. Continuous variables are expressed in terms of their means and standard deviations. Demographic characteristics and physical activity times of the subclinical depression and independent samples t-tests were used to compare the health control groups. Behavioural performance data were analysed using two-way mixed repeated-measures variance (ANOVA) analyses. A significance level of 0.05 was set for the two-sided p-value. Pearson correlation coefficient analysis was used for correlation analysis, including the correlation between BDI-II scores and physical activity time, as well as BDI-II scores and behavioural performance data. Mediation analysis was assessed using the bias-corrected nonparametric percentile Bootstrap method. Statistical analyses were conducted using SPSS 23.0 for Windows (SPSS, Inc., Chicago, IL, USA).

## 3 Results

### 3.1 Demographic results

The demographic information of the participants presented in **Table 1**. Between the healthy control and subclinical depression groups, there were no statistically significant differences in age, body mass index (BMI), or educational attainment (*p* > 0.05). However, notable distinctions were found in depressed mood, as evidenced by significantly higher BDI-II scores in the subclinical depression group compared to the healthy control group (*p*< 0.001).

**Table 1:** Demographic information of participants.

Groups	Age (years)	Height (cm)	Weight (kg)	BMI (kg/m^2^)	BDI-II
Healthy control group (n = 38)	18.72 ± 0.36^1^	162.7± 6.62	52.37 ± 4.72	20.79 ± 2.73	3.46 ± 0.73
ubclinical depression group (n = 40)	18.51 ± 0.42	160.7 ± 6.73	50.00 ± 1.92	19.43 ± 1.61	24.86 ± 2.02^2^

^1^Mean ± standard deviation, ^2^p< 0.001.

### 3.2 Physical activity time

**Figure 2:**
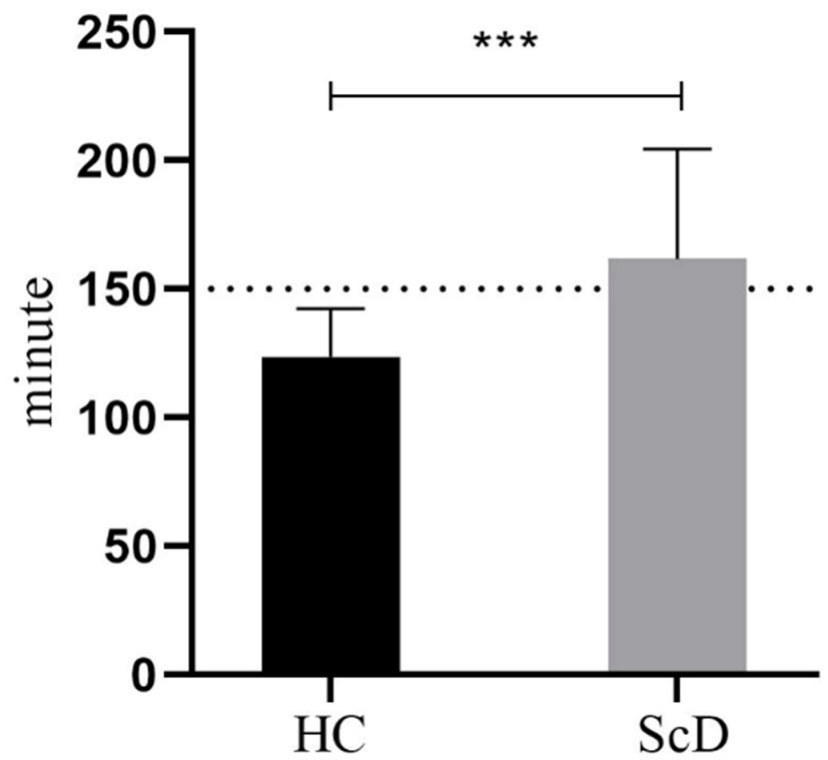
Moderate-intensity physical activity time of healthy control and subclinical depression groups. In the figure, HC (healthy control group), ScD (subclinical depression group), ***p < 0.001.

The Shapiro-Wilk analysis confirmed the normality of the physical activity time data. Importantly, the results revealed that moderate-intensity physical activity time was significantly lower in the subclinical depression group (123.69 ± 18.2 mins) compared to the healthy control group (161.77 ± 42.65 mins) (*t* = 5.17, *p*< 0.001, *Cohen’s d* = 1.16). The physical activity time results for the healthy control and subclinical depression groups are shown in **Figure 2**.

According to the WHO physical activity guidelines, adults should engage in 150–300 minutes of moderate-intensity physical activity per week (38). In our study, participants in the healthy control group met this recommendation by engaging in physical activity at a moderate level. However, the physical activity times of participants in the subclinical depression group fell short of the recommended 150 minutes of moderate-intensity physical activity per week.

### 3.3 Behavioural performance of executive function

**Figure 3:**
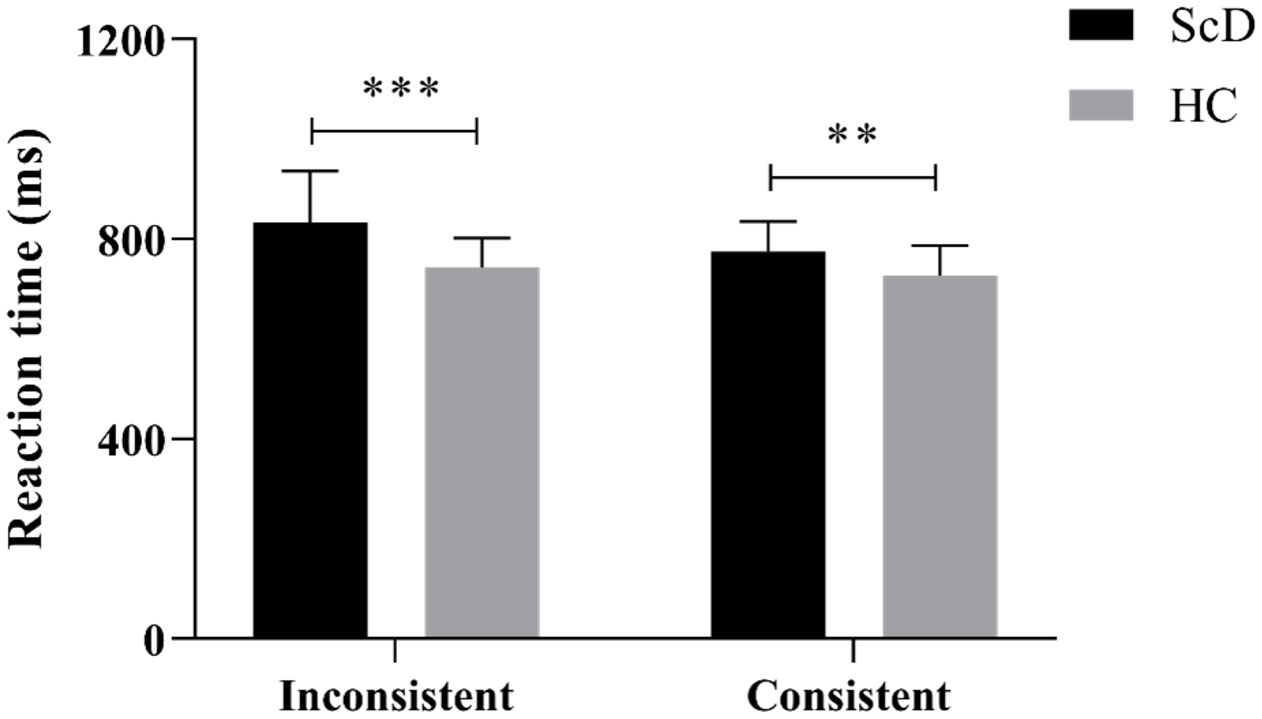
The reaction time of executive function in consistent and inconsistent conditions of healthy control and subclinical depression groups. In the figure, HC (healthy control group), ScD (subclinical depression group), **p < 0.01, ***p < 0.001.

A 2 (healthy control group vs subclinical depression group) × 2 (consistent vs inconsistent conditions) mixed repeated measures ANOVA was conducted on the reaction time and accuracy of executive function for the colour-word Stroop task. No outliers were identified in the box plots. The Shapiro-Wilk test indicated that the variables (reaction times and accuracy) followed a normal distribution (*p* > 0.05), and Levene’s test showed that the variances of variables in each group were equal (*p* > 0.05). The results of reaction times of executive function are displayed in **Figure 3**.

The results demonstrated a significant main effect of the colour-word condition, *F*(1, 76) = 29.38, *p*< 0.000, $\eta_{p}^{2\;}\;$ = 0.28. *Post hoc* analysis revealed that the reaction time was significantly longer for the colour-word inconsistent condition (685.38 ± 9.63 ms, 95% CL [666.20, 704.56]) compared to the colour-word consistent condition (639.55 ± 6.21 ms, 95% CL [627.18, 651.93], *p*< 0.001). The main effect of the group was also significant, *F*(1, 76) = 4.5, *p*< 0.000, $\eta_{p}^{2}$ = 0.28. *Post hoc* analysis indicated that the reaction time of the subclinical depression group (699.72 ± 9.65 ms, 95% CL [680.50, 718.94]) was significantly longer than that of the healthy control group (625.21 ± 9.90 ms, 95% CL [605.49, 644.94], *p*< 0.001).

Furthermore, there was a significant interaction between the colour-word conditions and groups, *F*(1, 76) = 29.03, *p* = 0.037, $\eta_{p}^{2\;}$ = 0.06. Simple effects analysis of the colour-word conditions revealed significant differences between the subclinical depression and healthy control groups in both the colour-word consistent and inconsistent conditions, all *p<* 0.001. Additional analyses indicated that the reaction time of the subclinical depression group was longer than that of the healthy control under the inconsistent condition (*t* = 4.80, *p*< 0.001, *Cohen’s d* = 0.48). Similarly, the reaction time of the subclinical depression group was longer than that of the healthy control group under the consistent condition (*t* = 3.59, *p*< 0.01, *Cohen’s d* = 0.34).

**Figure 4:**
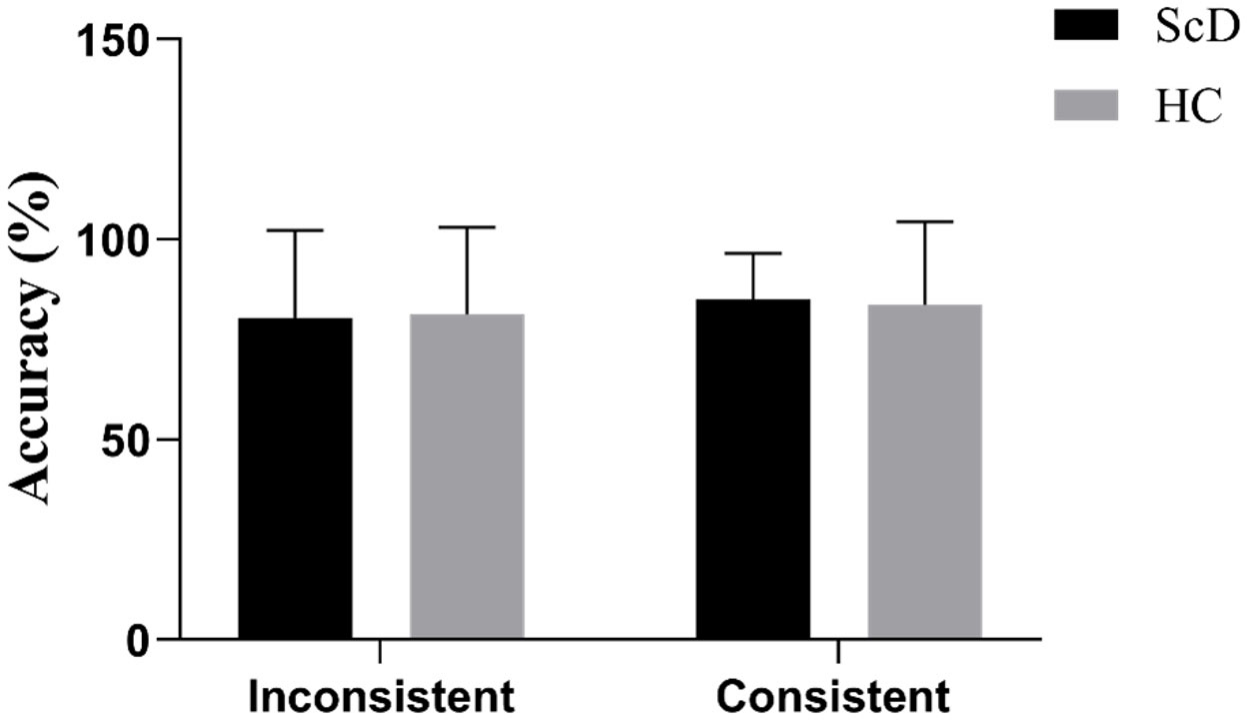
Accuracy of executive function in consistent and inconsistent conditions of healthy control and subclinical depression groups. HC (healthy control group), ScD (subclinical depression group).

Regarding accuracy, there was no significant difference between the subclinical depression and healthy control groups under both the consistent (81.25 ± 21.79% vs. 80.39 ± 21.86%) and inconsistent conditions (83.65 ± 20.77% vs. 85.07 ± 11.50%). The results of executive function performance accuracy are depicted in **Figure 4**.

### 3.4 Correlation and Mediation analysis results

The results of the correlation analysis revealed a significant negative correlation between the moderate-intensity physical activity time and BDI-II scores (*r* = -0.64; *p*< 0.001). Additionally, a significant positive association was found between the reaction time of executive function in inconsistent conditions and the BDI-II scores (*r* = 0.48; *p* = 0.002). Therefore, it can be concluded that for female college students with subclinical depression, only the time spent on moderate-intensity physical activity is negatively related to depression. Conversely, the reaction time of executive function in inconsistent conditions is positively correlated with depression.

Previous research has established a dose-response relationship between physical activity and depression. The correlation analysis in this study revealed a significant association among physical activity, executive function, and subclinical depression in female college students. To confirm whether executive function acts as a mediator between physical activity and subclinical depression, a bias-corrected nonparametric percentile Bootstrap method was employed. The mediated effects analysis demonstrated that moderate-intensity physical activity time significantly and positively predicted the reaction time of executive function in inconsistent conditions. When both moderate-intensity physical activity time and reaction time of executive function in inconsistent conditions were entered into the regression equation, both variables significantly and negatively predicted the level of depression in female college students. The bootstrap 95% confidence interval of the mediating effect did not include 0 ([-0.87, -0.03]), indicating that the mediating effect is significant (see **Table 2** for details).

**Table 2:** Results of mediation analysis.

Index	Physical activity time					Reaction time					BDI-II				
	*B* ^1^	*SE* ^2^	*t*	*p*	*β*	*B*	*SE*	*t*	*p*	*β*	*B*	*SE*	*t*	*p*	*β*
	33.038^3^	1.522	21.708	0.000	–	1082.795^3^	108.041	10.022	0.000	–	27.837^4^	2.767	10.059	0.000	–
Physical activity time	-0.072^3^	0.012	-5.921	0.000	-0.693	-2.1164	0.864	-2.449	0.019	-0.369	-0.062^4^	0.012	-4.961	0.000	-0.595
Reaction time											0.005^4^	0.002	2.206	0.034	0.265
*R²*	0.480					0.136					0.540				
Adjust *R²*	0.466					0.114					0.515				
*F*	*F* (1,38) = 35.055, *p* =0.000					*F* (1,38) = 5.998, *p* =0.019					*F* (2,37) = 21.745, *p* =0.000				

^1^Regression Coefficient, ^2^Standard Error, ^3^p< 0.01, ^4^p< 0.05.

**Figure 5:**
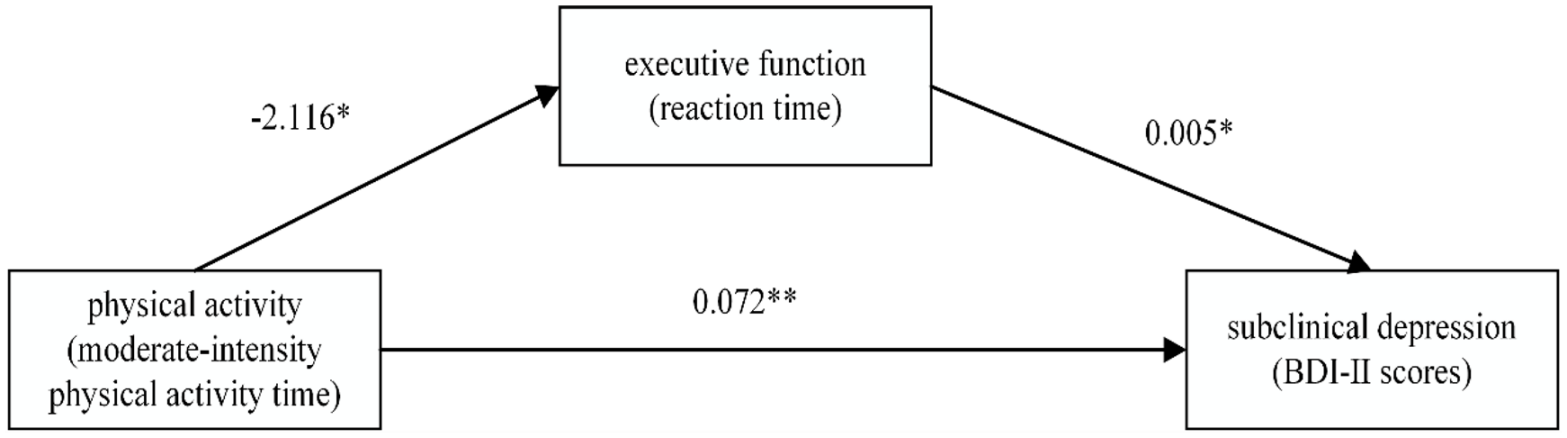
The mediating affection of executive function between physical activity and subclinical depression. In the figure, *p < 0.05, **p < 0.01.

Thus, executive function (specifically, the reaction time of colour-word Stroop under inconsistent conditions) partially mediates the relationship between physical activity (moderate-intensity physical activity time) and subclinical depression (BDI-II scores). The path diagram illustrating the mediating effect is shown in **Figure 5**.

From **Figure 5**, it can be observed that for female college students with subclinical de-pression, physical activity can influence executive function to a certain extent and im-prove depression through this mediation. Executive function has a partial mediating effect on the relationship between physical activity and subclinical depression.

## 4 Discussion

This study aimed to compare the physical activity and executive function of female college students with subclinical depression to those of healthy female college students. The goal was to explore the characteristics and correlation between physical activity and executive function in female college students with subclinical depression. Regarding physical activity time, the study found that the moderate-intensity physical activity time of female college students in the subclinical depression group was significantly lower than that of female college students in the healthy control group. Specifically, the moderate-intensity physical activity time of female college students with subclinical depression fell below the recommended standard of 150 minutes per week according to the physical activity guidelines of the WHO. This finding aligns with previous research on the relationship between physical activity and depression (40, 41). Therefore, it suggests that there is a correlation between depression and reduced physical activity time among female college students with subclinical depression.

In the test of behavioural performance of executive function, there was no significant difference between the subclinical depression group and the healthy group in terms of accuracy in completing the colour-word Stroop task. However, the reaction time of the subclinical depression group was significantly longer than that of the healthy group. This suggests that individuals in the subclinical depression group may experience greater difficulty or exert more effort when performing tasks that require executive functioning, even if they can accurately complete those tasks. According to the Stroop task, when a group takes longer or has a lower accuracy rate compared to others, it indicates that the group may struggle to complete the task (42). Therefore, the results of this study suggest that even mild depressive symptoms can impact cognitive performance and behaviour among female college students in the subclinical depression group, potentially leading to increased effort or inefficiency in task completion. Floros et al. (43), in a study on subclinical attention deficit hyperactivity disorder symptoms, also found evidence that cognitive decline caused by mild symptoms may initially manifest as changes in reaction times rather than errors. The study results indicate a potential impact of subclinical depression on both the physical activity time and executive function of female college students. Therefore, a correlation analysis was conducted on BDI-II scores, physical activity time, and behavioural performance of executive function in the subclinical depression group. The results revealed a negative correlation between BDI-II scores and moderate-intensity physical activity time in the subclinical depression group. This result is consistent with studies on patients with depression. A systematic review published by Roshanaei-Moghaddam et al. found that multiple studies have confirmed that baseline depression may lead to a sedentary lifestyle or decreased level of physical activity (40). Additionally, the reaction time for female college students with subclinical depression to complete the colour-word incongruent condition of the Stroop task was positively correlated with BDI-II scores. This result suggests that for female college students with subclinical depression, the level of their depressive symptoms may affect their executive function. This finding aligns with the perspective of some researchers who believe that depression has a detrimental impact on the performance of executive function in college students (15, 17, 18).

Finally, the mediation analysis (see **Figure 5**) indicates that among female college students with subclinical depression, a decrease in physical activity time may contribute to impaired executive function, consequently exacerbating subclinical depression. The mediation analysis results reveal that for female college students with subclinical depression, executive function played a partial mediating role in the relationship between physical activity and subclinical depression. A potential reason for the mediating role of executive function between physical activity and subclinical depression could be that appropriate physical activity enhances blood circulation, leading to increased oxygen supply to the brain and ultimately improving neural connections (44). In contrast, insufficient physical activity may weaken this physiological mechanism and impair the individual’s executive function.

Therefore, in a comparative study of female college students with subclinical depression and healthy female college students, the findings confirm that (1) for female college students, physical activity and executive function are associated with subclinical depression; (2) executive function serves as a partial mediator in the relationship between physical activity and subclinical depression. Hence, the impact of physical activity on the executive functions and depressive symptoms of female college students with subclinical depression remains a topic warranting further investigation, as suggested by the findings of this study. Additionally, research has confirmed that regular physical activity can have a positive effect on executive functions. Ludyga et al. (45) conducted a meta-analysis studying the acute effects of moderate aerobic exercise on specific aspects of executive function in different age and fitness groups. The results suggest that a single exercise session can influence executive function. Similarly, Shi et al. (46) demonstrated that moderate-intensity interventions, lasting 30–50 minutes at least three times per week for 17 weeks or more, have more beneficial effects on short- and long-term executive functions in children and adolescents.

However, it is essential to recognise several limitations when interpreting our findings. Firstly, all samples were drawn from the same university. Further verification is necessary to determine the generalizability of the conclusions of this study to female college students with subclinical depression in universities with diverse backgrounds. Secondly, this study is a cross-sectional study. Given that subclinical depression, physical activity, and executive function may change for individuals, longitudinal research is needed to explore the dynamic relationship among these three factors.

## 5 Conclusions

The results of the correlation analysis revealed a significant negative correlation between BDI-II scores and both physical activity time and executive function in female college students with subclinical depression. This suggests that as BDI-II scores increase, physical activity time decreases, and executive function performance tends to be poorer.

Furthermore, the mediation analysis provided additional insights into the relationship between physical activity, executive function, and subclinical depression. It revealed that executive function plays a partial mediating role in the association between physical activity and subclinical depression in female college students. In other words, the effect of physical activity on subclinical depression is partially explained by its impact on executive function.

These findings have important implications for understanding the interplay between physical activity, executive function, and subclinical depression. They suggest that both physical activity and executive function are relevant factors to consider when examining the mental well-being of female college students with subclinical depression. The negative correlation between BDI-II scores and physical activity time highlights the potential benefits of engaging in regular moderate-intensity physical activity for managing subclinical depression symptoms. Additionally, the mediating role of executive function suggests that interventions targeting executive function skills may be beneficial in improving mental health outcomes in this population.

## Data availability statement

The raw data supporting the conclusions of this article will be made available by the authors, without undue reservation.

## Ethics statement

The studies involving humans were approved by the Ethics Committee of Shaanxi Normal University (protocol code 202116010). The studies were conducted in accordance with the local legislation and institutional requirements. The participants provided their written informed consent to participate in this study.

## Author contributions

PL: Conceptualization, Funding acquisition, Investigation, Methodology, Writing – original draft. MMA: Formal analysis, Software, Writing – review & editing. HL: Data curation, Software, Writing – review & editing. HW: Conceptualization, Resources, Writing – review & editing. SZ: Conceptualization, Methodology, Project administration, Writing – review & editing.
